# A fatal case of acute encephalopathy in a child due to coxsackievirus A2 infection: a case report

**DOI:** 10.1186/s12879-021-06858-2

**Published:** 2021-11-18

**Authors:** Tomonori Nagai, Nozomu Hanaoka, Harutaka Katano, Masami Konagaya, Keiko Tanaka-Taya, Hiroyuki Shimizu, Toshiji Mukai, Tsuguto Fujimoto

**Affiliations:** 1 Department of Legal Medicine, ST. Marianna University School of Medicine, 2-16-1 Sugao, Miyamae-ku, Kawasaki-shi, Kanagawa, 216-8511 Japan; 2 Center for Emergency Preparedness and Response, National Institute of Infectious Diseases, 1-23-1 Toyama, Shinjuku-ku, Tokyo, 162-8640 Japan; 3 Department of Pathology, National Institute of Infectious Diseases, 1-23-1 Toyama, Shinjuku-ku, Tokyo, 162-8640 Japan; 4 Center for Surveillance, Immunization, and Epidemiologic Research, National Institute of Infectious Diseases, 1-23-1 Shinjuku-ku, Tokyo, 162-8640 Japan; 5 Department of Virology II, National Institute of Infectious Diseases, 4-7-1 Gakuen, Musashimurayama-shi, Tokyo, 208-0011 Japan

**Keywords:** Encephalopathy, Coxsackievirus A2, Sudden death, Autopsy, Case report

## Abstract

**Background:**

Certain types of enteroviruses, including coxsackieviruses, cause encephalitis, and other neurological complications. However, these pathogens rarely cause fatal infections, especially in immunocompetent infants. In this study, we present a rare case of acute encephalopathy caused by coxsackievirus A2 (CV-A2), which progressed rapidly in a previously healthy female child.

**Case presentation:**

In June 2013, a 26-month-old female child from Kanagawa, Japan, was found unresponsive during sleep. She was healthy until that morning. Her temperature was 37 °C at 08:00. She was feeling fine and went to the nursery that same morning. However, her condition worsened around noon. Therefore, she went home and slept at around 13:00. Surprisingly, after 2 h, her parents checked on her and found that she was lying on her back and was not breathing. Hence, she was immediately taken to a hospital by ambulance, but she was declared dead on arrival at the hospital. Subsequently, pathological autopsy and pathogenetic analysis, including multiple pathogen detection real-time PCR, were conducted to investigate the cause of death. The examination results revealed that she had an infectious respiratory disease and acute encephalopathy due to a CV-A2 infection.

**Conclusions:**

Based on our findings, we concluded that a previously healthy girl who had no immediate history of underlying medical condition were susceptible to death by acute encephalopathy due to CV-A2 infections. We proposed this conclusion because the patient’s condition progressed rapidly in less than 2 h and eventually led to her death. This is the first report on an acute encephalitis-dependent death in a child due to CV-A2 infection.

**Supplementary Information:**

The online version contains supplementary material available at 10.1186/s12879-021-06858-2.

## Background

Coxsackieviruses (CVs), which belong to the genus *Enterovirus* (EV), are divided into groups A (coxsackievirus A, CV-A) and B (coxsackievirus B, CV-B). The current classification of the EV consists of species A to D according to pathogenicity and molecular biologically. Enterovirus species A (EV-A) includes coxsackievirus A (CV-A) type 2–8, 10, 12, 14, 16. EV-B includes CV-A9, CV-B type 1–6. EV-C includes CV-A1, 11, 13, 17, 19, 20, 21, 22, and 24 [1]. CV infections are mostly benign with a good clinical prognosis, and infected patients generally respond well to symptomatic treatments alone [2].

According to the EV surveillance data from the American Center for Disease Control and Prevention [3], out of the 49,637 reported cases of EV infections, 3825 patients (7.7%) were infected with CV-A. Of these 3825 patients, 87 patients were infected with the CV-A2 strain (0.2%). Similarly, in Japan, a survey of 860 possible cases of EV infections in the third-largest municipality revealed that 105 patients (12.2%) were infected with CV-A, with only three patients infected with the CV-A2 strain (0.3%) [4]. The findings of this survey indicated that CV-A2 infections were rare among EV infections. Nevertheless, EV-A71 and other EVs cause encephalitis and other neurological complications [5]. Furthermore, although neurological involvement following a CV-A2 infection is infrequent, few CV-A2 infected patients have shown altered consciousness and acute-flaccid paralysis [6], and fatal myocarditis [7]. Additionally, in Hong Kong, four children infected with CV-A2 showed severe respiratory symptoms, and two of these four children died [8]. However, there are no reports on fatal encephalitis cases with CV-A2.

Therefore, this report presents the autopsy and pathogenetic analysis of a sporadic, virologically confirmed fatal case of CV-A2-induced acute encephalopathy.

## Case presentation

The deceased was a 26-month-old female child living with her parents and two brothers in Kanagawa, Japan. She had no known growth or developmental abnormalities and no relevant medical history, including neurological and immunological diseases.

In June 2013 at 8:00 a.m., she was fine with a body temperature of 37 °C and she went to the nursery. However, by 11:30, she started feeling unwell and her father brought her back home. At 13:40, she was observed to be sleeping on her back. Then, around 15:00, she was found lying with her face down, immobile, and not breathing. At this point, she was immediately taken to a hospital. Her body temperature was 36.6 °C when the ambulance service team checked her, but she was declared dead on arrival at the hospital. Rigor mortis had already set in at the temporomandibular joints. From our analysis, the time of death was estimated to be approximately 14:00 based on the degree of rigor mortis. After obtaining permission from police officials, an autopsy was conducted 2 days after death.

**Fig. 1:**
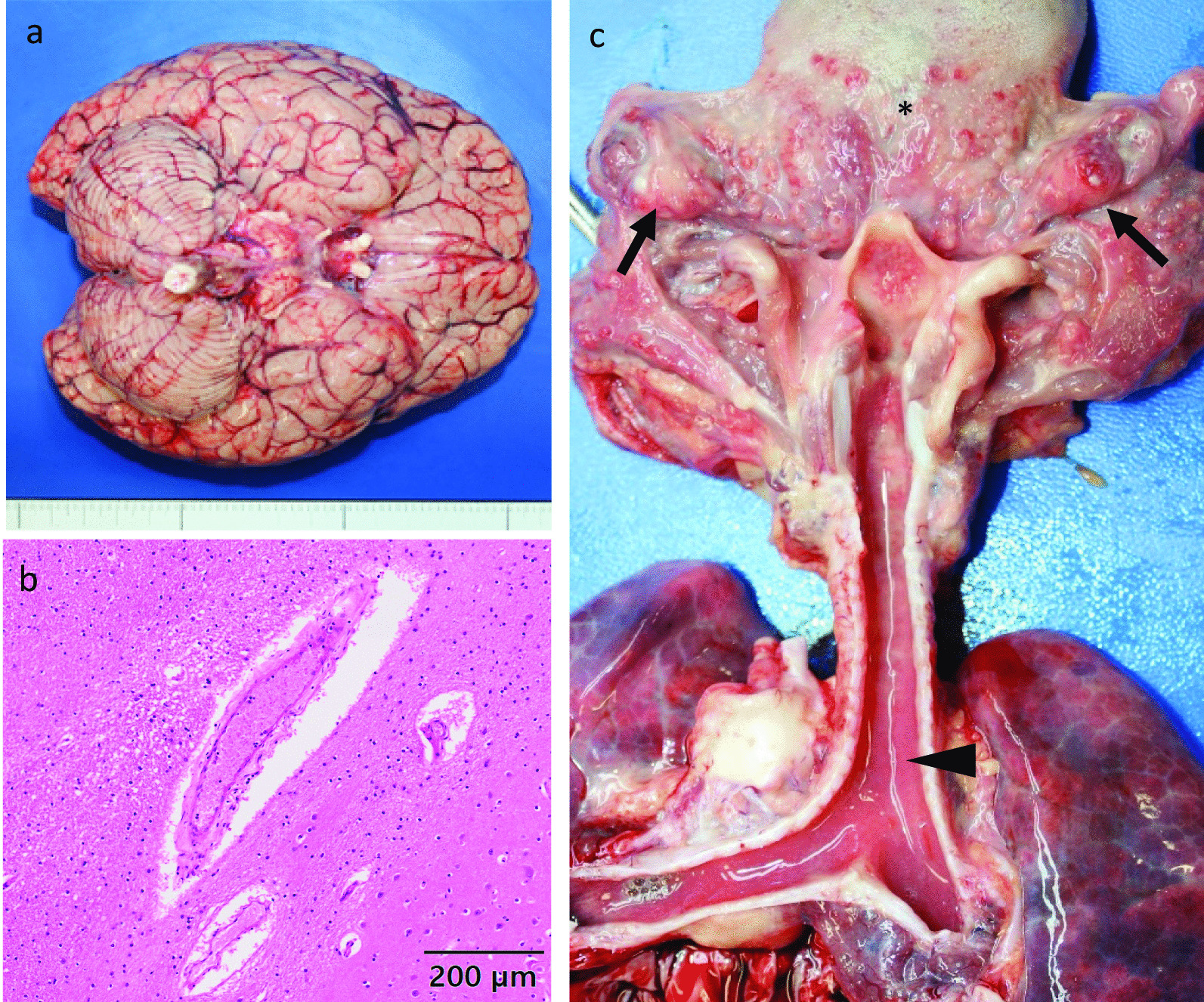
Pathological findings in the brain (**a**, **b**) and upper respiratory tract (**c**). **a** Inferior view of the brain. No macroscopic abnormalities were observed. **b** Hematoxylin and eosin staining of the brain. Some perivascular extravasation of serum proteins and dilatation of Virchow Robin spaces are found without inflammatory cell infiltration. Original magnification x100. Scale bar = 200 μm. Microscopic images were taken with VS200 Slide Scanner (Olympus, Tokyo, Japan) at 0.274 μm/pixel, and analyzed with Olyvia software (Olympus). The image was not edited. **b** Upper respiratory tract. Redness was observed on the palatine tonsils (arrow), lingual tonsils (*), and the entire tracheal and bronchial mucosa (arrowhead)

The pathological postmortem and histological findings are shown in Fig. 1. Microscopic images were taken with VS200 Slide Scanner (Olympus, Tokyo, Japan) at 0.274 μm/pixel, and analyzed with Olyvia software (Olympus). Her height and weight were 89.5 cm and 12 kg, respectively. The skin was normal and no rashes or signs of trauma were observed. An intense purplish-red postmortem lividity had developed. Profound rigor mortis had also set at the joints. The brain weighed 1090 g, and although considerable cerebral edema was observed, no obvious space-occupying lesions or cerebral contusions were present. Severe laryngopharyngeal congestion, swelling of the palatine tonsils, and redness around the lingual tonsils were observed as well. Similarly, the tracheal and bronchial mucosae were severely congested; however, no evidence of pneumonia was found. Swelling of the peritracheal and mesenteric lymph nodes was also observed. However, there was no evidence of herpangina-like blisters or ulcers in the oral cavity. Furthermore, no congenital malformations or morbid abnormalities in the cardiovascular system, and importantly, no macroscopic and histological abnormalities were observed in any other organ. Therefore, acute encephalopathy due to viral respiratory infection was suspected as the cause of death.

Because we are studying the pathogenicity of EVs, we first performed a specific test for enteroviruses. Throat and nasal swabs, as well as feces, cerebrospinal fluid, serum, and urine samples, were analyzed using RT-PCR. Consensus-degenerate hybrid oligonucleotide primers [9] were used to amplify a part of the EV viral protein (VP) 1 gene. All samples, except the cerebrospinal fluid and urine samples, were RT-PCR-positive for EV. Based on the results of the analysis using the basic local alignment search tool (BLAST) [10], the detected EV was typed CV-A2 because it had more than 80% nucleotide and 95% amino acid identity with the prototype CV-A2 strain (Fleetwood, AY421760.1). The sequence was deposited and available in INSD (the International Nucleotide Sequence Databases) (accession number LC656263.1).

Then, the cerebrospinal fluid and urine samples were further analyzed. We conducted RT-PCR using two sets of specific primers (Table 1) designed to detect and sequence the VP1 region of the CV-A2 strain. After using random hexamers to synthesize the cDNA, we conducted an initial RT-PCR to amplify the 217-bp fragment using the primers CA2_F1 and CA2_R1. Subsequently, another PCR was conducted to amplify the 201-bp fragment using the primers CA2_F2 and CA2_R2. Consequently, the cerebrospinal fluid sample tested positive for the presence of CV-A2, whereas the urine sample tested negative. Moreover, when the base sequences from the cerebrospinal fluid were compared with the sequence of the strain 431135 (JX867332.1), a 96.2% (151/157 bp) homology was observed (Table 2). All VP1 sequences determined in this study were identical. Phylogenetic analysis identified the strain as CV-A2 (Additional file 3: Fig. S1).

**Table 1:** Specific primers used in this study

Primer	Sequences (5′→3′)	Polarity	Position^a^		Identity	%
CA2_F1	TGTGTGGTTAACAAAAATAGTG	Forward	2643–2664		22/22	100
CA2_R1	TGGTAGCCACAAACGTGAACTC	Reverse	2859–2838		22/22	100
CA2_F2	TAACAAAAATAGTGTGGAAGAGG	Forward	2651–2673		23/23	100
CA2_R2	ACAAACGTGAACTCAGCATTG	Reverse	2851–2831		21/21	100

^a^VP1 region of CV-A2 at strain 431135 (JX867332.1)

**Table 2:** Summary of CV-A2 detections in various tissue samples

Clinical samples	CODEHOP RT-PCR	CV-A2 specific RT-PCR	Identity^a^ (%)
Pharyngeal swab	+	Not done	213/219 (97.3)
Throat swab	+	Not done	213/219 (97.3)
Cerebrospinal fluid	−	+	151/157 (96.2)
Feces	+	Not done	213/219 (97.3)
Serum	+	Not done	213/219 (97.3)
Urine	−	−	−

+ means positive for RT-PCR. − means negative for RT-PCR

^a^VP1 region of CV-A2 at strain 431135 (JX867332.1) position 2643–2861 for CODEHOP RT-PCR, and position 2674–2830 for CV-A2 specific RT-PCR

Therefore, based on the results described above, we attempted to directly detect the virus in various organs to provide an accurate diagnosis. Nucleic acids were extracted from the paraffin sections of each organ and the cryopreserved organs (heart, liver, and kidney). We first performed a comprehensive viral survey using a multivirus real-time PCR to simultaneously detect 167 viruses (Additional file 1: Table S1) [11]. Pan-EV and cytomegalovirus (CMV: Human herpesvirus-5) were detected from the paraffin sections at this step. Next, real-time RT-PCRs for pan-EV and CMV were performed using nucleic acids extracted from the paraffin sections of individual organs (Additional file 2: Table S2). Some brain sections were found to be EV and CMV positive, although the copy number was low. Frozen samples from the heart, liver, kidney, as well as brain, were CMV positive. CMV was detected in a paraffin section sample of the brain and frozen samples from the heart, liver, and kidney. However, copy numbers of CMV were low and no positive signal was detected in immunohistochemistry for CMV in the brain and heart. CMV was detected in the kidney, although CMV is frequently detected in the kidney of healthy persons [12]. Thus, these data strongly suggest that CMV infection was not the main cause of death.

Individual tissues and paraffin sections from each organ were then immunostained using a previously reported in-house anti-EV rabbit polyclonal antibody [13]. Results showed no clear immune reactivity (data not shown). This result and a positive but trace amount of EV detected from the brain section were proposed to be associated with acute encephalopathy (not encephalitis).

Finally, acute encephalopathy due to CV-A2 infection was diagnosed based on the following observations: (a) CV-A2 was detected in the throat and nasal swabs, serum, feces, and cerebrospinal fluid, and (b) clear signs of encephalopathy, including cerebral edema, were observed despite the absence of inflammatory cell infiltration in the brain. In addition, no evidence of inflammation, such as pneumonia or myocarditis, and no obvious abnormalities in any other organ were found. A previous study reported that influenza virus-induced acute encephalopathy does not show extensive inflammation, and the virus cannot be directly detected in the brain [14]. In the present case, a cytokine storm due to CV-A2-induced acute encephalopathy is proposed to have led to the child’s sudden death.

## Discussion and conclusions

Based on the findings of the pathological autopsy and the molecular genetic analysis, we diagnosed the cause of the sudden death of a 26-month-old girl as acute encephalopathy due to CV-A2 infection. In recent years, by using sensitive RT-PCR methods which allow the detection of a range of enteroviruses, it has been reported that CV-A is involved in meningitis and acute encephalopathy. Acute encephalopathy caused by CV-A2 is rare as the number of infected patients is small [3, 4]. This is the first report on acute encephalitis-dependent death in a child due to CV-A2.

Therefore, as a preventive public health measure in combating acute encephalopathy, CV-A2 should be considered one of the putative causative agents.

## Supplementary Information

**Additionalfile 1: Table S1.** Results of multivirus real-time PCR to simultaneously detect 167 viruses. Pan-EV and cytomegalovirus (CMV: Humanherpesvirus-5) were detected from the paraffin section.

**Additionalfile 2: Table S2.** Results of Enterovirus and Cytomegalovirus detection. Some of the brain sections were found to be EV and CMV positive, although the copy number was low. Copy numbers per approximately 100 ng of DNA or RNA are shown. The unit of β-actin was copy per approximately 100 ng of DNA or RNA.

**Additionalfile 3: Fig. S1.** Phylogenetic analysis of detected sequences with Coxsackie Virus A2 and A4. Evolutionary relationships of the partial VP1 sequence amplified with CODEHOP primers (A) and partial VP1 sequence amplifiedwith CV-A2 specific primers (B) were shown. The evolutionary history was inferred using the Neighbor-Joining method. The percentage of replicate treesin which the associated taxa clustered together in the bootstrap test (1000 replicates) are shown next to the branches. The tree is drawn to scale, with branch lengths in the same units as those of the evolutionary distances used to infer the phylogenetic tree. The evolutionary distances were computed using the Kimura 2-parameter method and are in the units of the number of base substitutions per site. The analysis involved 32 nucleotide sequences. Codon positions included were 1st + 2nd + 3rd + Noncoding. All positions containing gaps and missing data were eliminated. There were a total of 311(A), 108(B)positions in the final dataset. Evolutionary analyses were conducted in MEGA7 (https://www.megasoftware.net/). The white circle indicates the fatal myocarditis case reported by Bendig et al. [7]. The triangle indicates fatal pneumonia cases reported by Yip et al. [8]. The black circle indicates the detected sequence in this study.

## Data Availability

All data generated during this study are included in this published article and its Additional files. The datasets generated and/or analysed during the current study are open and available in the INSD under the accession numbers: AY421760.1, AY421762.1, HQ728259.1, JX867330.1, JX867331.1, JX867332.1, JX867333.1, KP289357.1, KP289358.1, KP289359.1, KP289360.1, KP289361.1, KX156350.1, KX156360.1, KX595281, KX595282.1, KX595283.1, KX595284.1, KX810065.1, L28146.1, LC656263.1, LR027549.1, LR027551.1, MF281257.1, MF678310.1, MF678322.1, MF678333.1, MF678334.1, MF678338.1, MG214257.1, and NC_038306.1.

## Abbreviations

CV: Coxsackieviruses
EV: Enterovirus
CV-A: Coxsackievirus A
CV-B: Coxsackievirus B
CODEHOP: Consensus-degenerate hybrid oligonucleotide primers
RT-PCR: Reverse transcription-polymerase chain reaction
VP1: Viral protein 1
CMV: Cytomegalovirus
