# Supplementary figures and images for: A fatal case of acute encephalopathy in a child due to coxsackievirus A2 infection: a case report
Source: BMC Infect Dis. 2021 Nov 18;21:1167. doi: 10.1186/s12879-021-06858-2 (PMC8600809; doi:10.1186/s12879-021-06858-2)

A

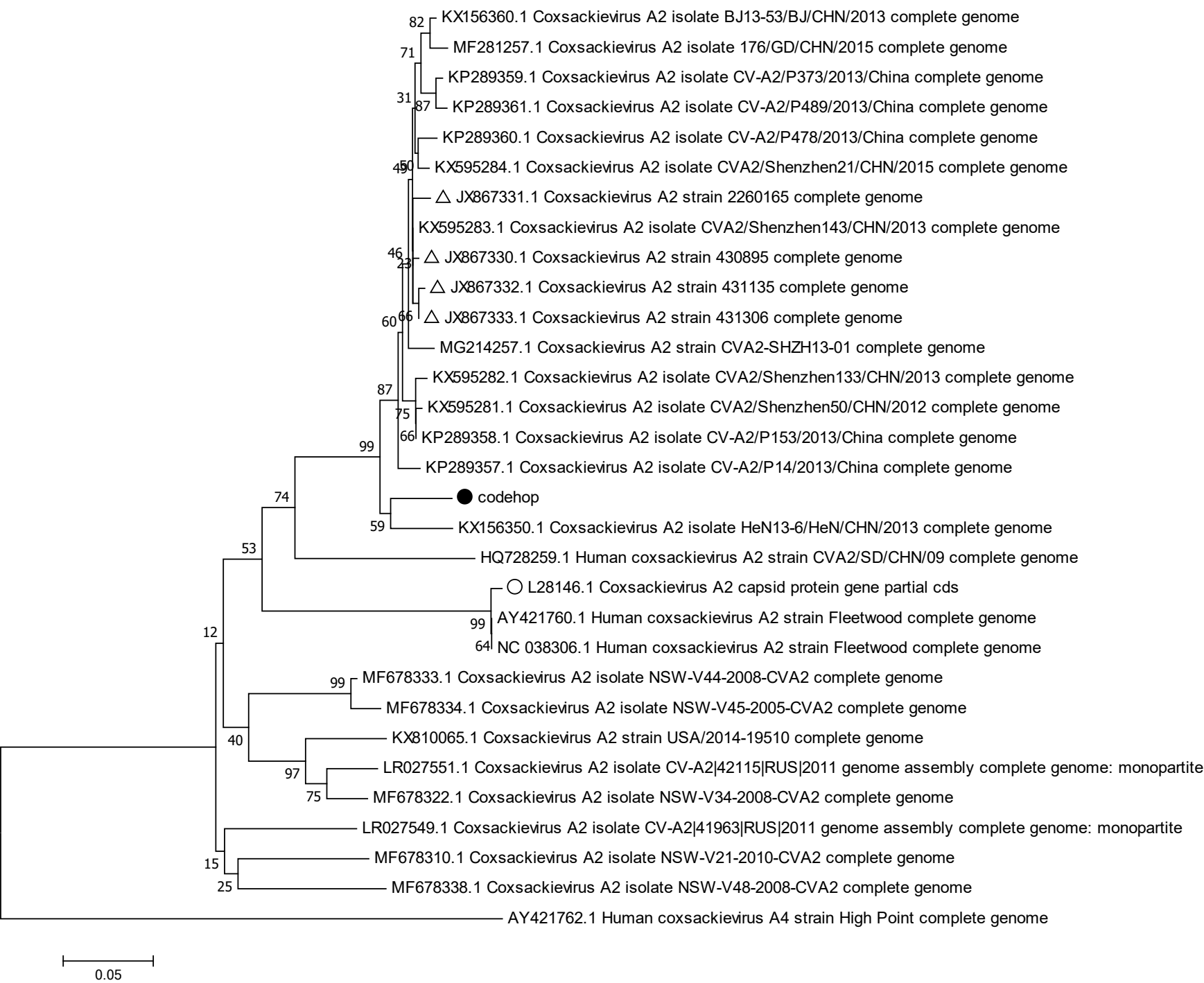

B

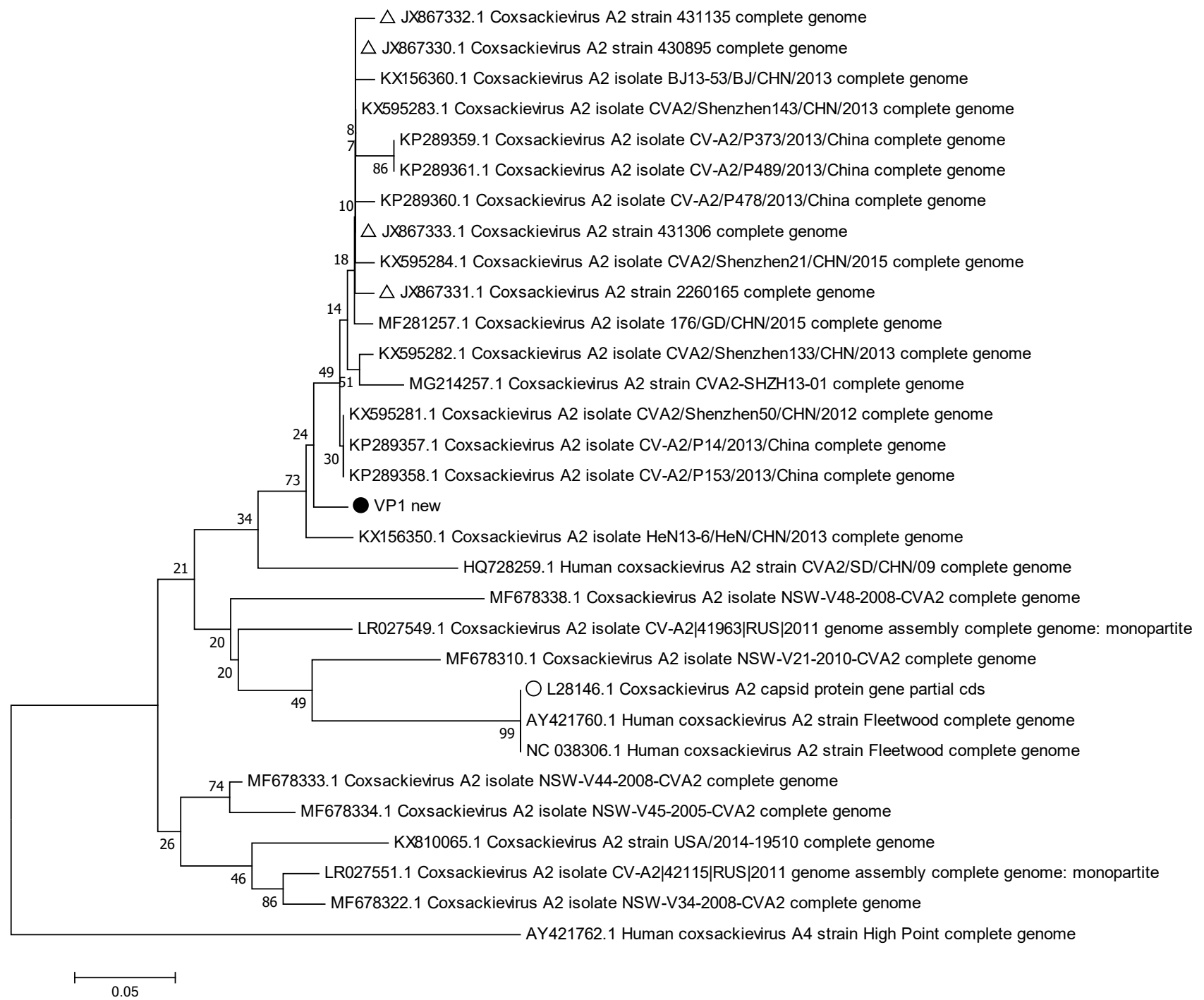

Supplement: Supplementary file 3 — Additionalfile 3: Fig. S1. Phylogenetic analysis of detected sequences with Coxsackie Virus A2 and A4. Evolutionary relationships of the partial VP1 sequence amplified with CODEHOP primers (A) and partial VP1 sequence amplifiedwith CV-A2 specific primers (B) were shown. The evolutionary history was inferred using the Neighbor-Joining method. The percentage of replicate treesin which the associated taxa clustered together in the bootstrap test (1000 replicates) are shown next to the branches. The tree is drawn to scale, with branch lengths in the same units as those of the evolutionary distances used to infer the phylogenetic tree. The evolutionary distances were computed using the Kimura 2-parameter method and are in the units of the number of base substitutions per site. The analysis involved 32 nucleotide sequences. Codon positions included were 1st + 2nd + 3rd + Noncoding. All positions containing gaps and missing data were eliminated. There were a total of 311(A), 108(B)positions in the final dataset. Evolutionary analyses were conducted in MEGA7 (https://www.megasoftware.net/). The white circle indicates the fatal myocarditis case reported by Bendig et al. [7]. The triangle indicates fatal pneumonia cases reported by Yip et al. [8]. The black circle indicates the detected sequence in this study. [file 12879_2021_6858_MOESM3_ESM.pdf]
